# Maternal and neonatal outcomes of Kielland’s rotational forceps-assisted vaginal births in a tertiary care center

**DOI:** 10.1016/j.xagr.2026.100708

**Published:** 2026-10-17

**Authors:** Paul Naseef, Caroline Leps, Elham Almoi, Daniel Kane, Nancy Kumar, Melissa Walker, John Kingdom, Sebastian Hobson

**Affiliations:** 1 Department of Obstetrics and Gynecology (Naseef, Leps, and Kumar), University of Toronto, Toronto, Canada; 2 Temerty Faculty of Medicine (Almoi), University of Toronto, Toronto, Canada; 3 Department of Obstetrics and Gynecology, Mount Sinai Hospital (Kane, Walker, Kingdom, Hobson), University of Toronto, Toronto, Canada

**Keywords:** assisted vaginal birth, cesarean section, Kielland’s rotational forceps, second stage labor dystocia

## Abstract

**BACKGROUND:**

Rates of second-stage Cesarean section (CS) have increased substantially, contributing to higher maternal morbidity and long-term risks in future pregnancies, including repeat CS and placenta accreta spectrum. Reducing unnecessary second-stage CS is therefore an important goal in modern obstetric care. Fetal malposition, particularly occiput transverse and posterior positions, is a key cause of labor dystocia and operative delivery. While several interventions exist, their success is variable. Kielland’s rotational forceps can facilitate vaginal birth and potentially avoid CS, but their use has declined due to safety concerns and limited training, leaving a gap in contemporary evidence comparing outcomes with other delivery methods.

**OBJECTIVE:**

To compare maternal and neonatal outcomes between rotational and nonrotational forceps use in a tertiary care setting.

**STUDY DESIGN:**

A retrospective cohort study was conducted of all forceps-assisted births at a tertiary care hospital in Toronto, between January 2014 and December 2021. Maternal outcomes included postpartum hemorrhage defined as estimated blood loss of 500 mL or more, and significant obstetric anal sphincter injuries defined as 3B, 3C and fourth degree perineal lacerations. Neonatal outcomes included Apgar scores, cord blood gases, need for resuscitation, and neonatal head trauma.

**RESULTS:**

Among 1606 forceps-assisted births, 26 (1.6%) required Kielland’s rotational forceps. Episiotomy was universal in the rotational group (n=26/26, 100%) compared with 90.1% (n=1423/1580) in the nonrotational group. Significant OASIS occurred less frequently with rotational forceps (7.6%, n=2/26 vs 10.8%, n=172/1580), while rates of PPH were comparable (26.9%, n=7/26 vs 28.8%, n=456/1580). No major PPH (≥1000 mL) or shoulder dystocia occurred in the rotational group. Low 1-minute Apgar scores (<7) were more common in the rotational group (26.9%, n=7/26 vs 14%, n=227/1580), but all infants achieved scores ≥7 at 5 minutes. No cases of cephalohematoma, subgaleal hematoma, or intracranial hemorrhage occurred in the rotational group.

**CONCLUSION:**

Kielland’s rotational forceps are a safe alternative to second-stage CS when performed by experienced operators. Their use was not associated with excess major maternal morbidity or significant neonatal trauma, in comparison to nonrotational forceps. Our data support efforts to transfer these skills to trainees and larger studies are needed to confirm these single institution findings.

**AJOG Global Reports at a Glance**

**Why was this study conducted?**

To address limited contemporary data on the safety of Kielland’s rotational forceps compared with non-rotational forceps in modern obstetric practice. The decline in operative vaginal delivery, together with increasing rates of second-stage Cesarean delivery, underscores the importance of maintaining appropriate expertise in operative vaginal delivery, including rotational forceps techniques.

**Key findings**

Rotational forceps were not associated with increased maternal or neonatal morbidity, with no major neonatal trauma observed despite more complex clinical scenarios.

**What does this study add to what is known?**

Provides real-world, contemporary evidence supporting the selective and skilled use of rotational forceps, while highlighting the need for larger, adjusted comparative studies.

## Introduction

Over the past decades, there has been a marked increase in the rate of cesarean section (CS) performed during the second stage of labor^1^ leading to a corresponding rise in maternal morbidity^2^^,^^3^ with a subsequent increased risk of repeat CS in subsequent pregnancies, placenta accreta spectrum disorders, uterine rupture, and preterm labor and birth.^4^^,^^5^

Malposition of the fetal head, particularly persistent occiput transverse (OT) or occiput posterior (OP) position, is a primary driver of second-stage labor dystocia, complicating up to 20% of labors and substantially increasing the risk of subsequent interventions.^6^^,^^7^ While expectant management, manual rotation of the fetal head or augmentation with oxytocin may resolve some of the cases, a significant proportion will require operative delivery, either by assisted vaginal birth (AVB) or CS, to mitigate the risks of prolonged labor, such as chorioamnionitis, fetal acidemia, and maternal exhaustion.^8^

For fetal malposition, options include manual rotation, nonrotational forceps, rotational forceps, or auto-rotational vacuum-assisted birth; however, vacuum extraction is limited for definitive rotation from transverse or posterior positions, with a higher risk of unsuccessful assisted vaginal birth.^8^ Manual rotation is a valuable skill, but it has a variable success rate, often dependent on the operator's experience as well as maternal and fetal factors.^9^ Nonrotational forceps deliveries in the occiput posterior position carry high OASIS rates (43%–72%), compared with lower rates (24%–33%) when rotation to the occiput anterior position is achieved.^10^

Kielland’s rotational forceps, invented in 1915, are designed for correcting deep transverse arrest and persistent fetal malposition, using a minimal pelvic curve and sliding lock to allow skilled rotation and safe extraction of the fetal head.^11^ When used by experienced clinicians, Kielland’s forceps can enable safe vaginal birth and avoid second-stage CS morbidities, but their use has declined due to concerns about maternal and neonatal trauma, reduced training, and medicolegal risks.^12^^,^^13^

This decline has created a critical knowledge gap and a clinical paradox.^14^ Despite the clear benefits of avoiding high-morbidity CS, contemporary data on the safety of Kielland’s forceps in modern tertiary care settings are limited, with most literature being outdated or based on small, single-center studies.^15^ There is a lack of robust comparative data on maternal and neonatal outcomes for rotational forceps births vs nonrotational forceps and, importantly, vs second-stage CS.

This study aimed to descriptively compare maternal and neonatal outcomes of Kielland’s rotational forceps deliveries with those of nonrotational forceps deliveries performed within the same institution.

## Methods

This study be regarded as descriptive and hypothesis-generating rather than as definitive evidence of equivalence or safety. It is a single-center retrospective cohort study at a Canadian tertiary care hospital examined all singleton forceps-assisted vaginal births from 2014–2021, with ethics approval (REB No. 2025-0724-1490). Data on maternal, neonatal, and operator-specific outcomes were extracted from electronic records. Exclusion criteria included gestation <35 weeks, multiple gestation, and assisted vaginal birth attempts that were converted to Cesarean delivery.

Data collected included AVB indications, instruments used, and fetal station/position at delivery. Neonatal outcomes recorded were cephalohematoma, subgaleal hematoma, skull fracture, Apgar scores, cord pH, and any need for resuscitation, confirmed by clinical assessment and imaging when indicated.

Maternal outcomes included episiotomy, perineal laceration severity, and obstetric anal sphincter injuries (OASIS). Postpartum hemorrhage was defined as blood loss >500 mL, and shoulder dystocia was recorded if specific maneuvers were required to deliver the shoulders. The Scanzoni maneuver was not utilized in the cohort described in this study.

Given the retrospective and nonrandomized nature of operative instrument selection, clinically relevant maternal and intrapartum characteristics such as maternal age, body mass index, comorbidities, birthweight, fetal position, and duration of the second stage of labor were not consistently available for analysis. Consequently, adjustment for potential confounding variables was not performed, and all comparisons presented are crude estimates of association. These findings should therefore be interpreted as descriptive rather than causal.

Categorical variables were compared using chi-square or Fisher’s exact tests as appropriate. Crude odds ratios (OR) with 95% confidence intervals (CI) were calculated. Where zero-cell counts occurred, a Haldane–Anscombe continuity correction was applied. All analyses were exploratory and hypothesis-generating.

## Results

Of 1606 forceps-assisted vaginal births, 1.6% (26/1606) required Kielland’s rotational forceps, mostly exclusively (88.5%), with a few followed by Simpson traction (11.5%). Nulliparity was 57.7% in the rotational group vs 63.3% in the non-rotational group. Gestational ages were similar (35–41+2 weeks vs 35–41+6 weeks). All rotational forceps births were mid-cavity, whereas nonrotational deliveries included mid-cavity (29.5%), low (57.6%), and outlet (6.8%) births as shown in Table 1.

**Table 1:** Type of forceps delivery by fetal station

	Rotational forceps % (n=26)	Nonrotational forceps % (n=1580)
Mid-cavity^a^	100% (n=26)	29.5% (n=467)
Low^b^	0%	57.6% (n=911)
Outlet^c^	0%	6.8% (n=109)

a Mid-cavity: when the fetal head is at station 0 (ischial spine) to +2 cm from the ischial spine

b Low: when the fetal head is lower than station +2 cm from the ischial spine but not yet visible at the introitus

c Outlet: when the fetal head is visible at the introitus (at station +4 cm or +5 cm from the ischial spine).

The most frequent indication for rotational forceps AVB was a nonprogressive second stage of labor, occurring in 57% (n=15/26) of cases, compared to 34.9% (552/1580) of the nonrotational cohort. This suggests a greater reliance on Kielland’s forceps in scenarios involving deep transverse arrest or failure of descent, consistent with their utility in facilitating rotation in malpositioned fetal heads.

Fetal compromise, suggested by atypical or abnormal fetal heart rate tracing, was also a major contributing indication in 53.8% (n=14/26) of rotational births, similar to 59.2% (n=935/1580) of nonrotational births. While overall nonreassuring, fetal heart rate tracing was a common indication across both groups, it did not disproportionately drive the use of Kielland’s forceps.

None of the rotational forceps-assisted births occurred in patients identified as having ineffective pushing in the second stage, whereas 9 nonrotational cases (0.6%) were performed for that reason.

Maternal medical indications, including conditions limiting Valsalva or prolonged pushing, were rare reasons (7.7% in rotational vs 5% in nonrotational). No rotational births occurred for maternal complications like hemorrhage or chorioamnionitis, and only one nonrotational case involved suspected placental abruption.

No cases of rotational forceps delivery were documented as being based on maternal request or ineffective analgesia, compared to a combined total of 4 such cases (0.24%) in the nonrotational group. The indications of AVB in both groups are summarized in Table 2.

**Table 2:** Indications for forceps AVB by type of forceps delivery

	Rotational forceps % (n=26)	Nonrotational forceps % (n=1580)	OR	95% CI
Nonprogressive second stage of labor	15 (57.7%)	552 (34.9%)	2.54	1.16–5.57
Abnormal/atypical FHR	14 (53.8%)	935 (59.2%)	0.8	0.37–1.75
Maternal medical condition	2 (7.7%)	79 (5%)	1.58	0.08–6.83
Ineffective maternal pushing	0 (0%)	9 (0.6%)	0	0–32.32
Placental abruption/antepartum hemorrhage	0 (0%)	1 (0.06%)	0	0–32.32
Maternal request	0 (0%)	1 (0.06%)	0	0–2370
Inadequate analgesia or pain relief	0 (0%)	3 (0.18%)	0	0–2370

Maternal outcomes are summarized in Table 3. Episiotomy was performed in all patients (100%, n=26/26) undergoing rotational forceps, compared to 90.1% (n=1423/1580) in the nonrotational forceps cohort. The overall incidence of obstetric anal sphincter injuries was lower among patients delivered with rotational forceps (7.6%; n=2) compared to those delivered with nonrotational forceps (10.8%; n=172).

**Table 3:** Maternal outcomes by type of forceps AVB

	Rotational forceps % (n=26)	Nonrotational forceps % (n=1580)	OR	95% CI
Episiotomy	100% (n=26)	90.1% (n=1423)	5.86	0.36–96.7
OASIS	7.6% (n=2)	10.8% (n=172)	0.68	0.16–2.91
PPH (≥500 mL)	26.9% (n=7)	28.8% (n=456)	0.91	0.38–2.18
PPH (≥1000 mL)	0% (n=0)	4.8% (n=76)	0.37	0.02–6.15
Shoulder dystocia	0% (n=0)	4.3% (n=69)	0.41	0.02–6.80

*OASIS*, obstetric anal sphincter injuries classified as third- or fourth-degree perineal lacerations; *PPH*, postpartum hemorrhage.

The rate of postpartum hemorrhage (PPH) did not significantly differ between both groups, occurring in 26.9% (n=7/26) of the rotational group vs 28.8% (n=456/1580) of the nonrotational group. Notably, no cases of PPH ≥1000 mL occurred in the rotational group, whereas it occurred in 4.8% (n=76/1580) of the nonrotational forceps births.

Shoulder dystocia was not observed in any rotational forceps cases, compared to a 4.3% incidence (n=69/1580) in the nonrotational group.

Although point estimates differed between groups, all comparisons were associated with wide confidence intervals that included clinically important harm and benefit. The 1-minute Apgar scores <7 represented 26.9% in the rotational group vs 14.1% in the nonrotational group. All rotational group neonates reached ≥7 by 5 minutes, compared with 98.2% in the non-rotational group as shown in Table 4.

**Table 4:** Neonatal Apgar scores and resuscitation requirement

	Rotational forceps % (n=26)	Nonrotational forceps % (n=1580)	OR	95% CI
Apgar score <7 at 1 min	26.9% (n=7)	14.4% (n=227)	2.2	0.91–5.28
Apgar score <7 at 5 min	0% (n=0)	1.8% (n=29)	0.99	0.06–16.67
Resuscitation required	11.5% (n=3)	12.6% (n=200)	0.9	0.27–3.02

Umbilical arterial cord pH <7.2 occurred in 11.5% of rotational and 10.6% of nonrotational births. Moreover, umbilical arterial cord pH <7.1 occurred only in 3.7% of rotational and 4.8% of nonrotational births. Neonatal resuscitation was required in 11.5% of rotational and 12.6% of nonrotational cases, showing similar rates between groups.

No traumatic cranial injuries occurred in the rotational forceps group, whereas in nonrotational forceps births, cephalohematoma occurred in 0.6%, subgaleal hematoma in 0.4%, and skull fractures with intracranial hemorrhage in 0.3% of cases as shown in Table 5.

**Table 5:** Neonatal blood gases and injury outcomes

	Rotational forceps % (n=26)	Nonrotational forceps % (n=1580)	OR	95% CI
Umbilical arterial pH <7.2	11.5% (n=3)	10.6% (n=169)	1.09	0.32–3.67
Cephalohematoma	0% (n=0)	0.6% (n=11)	2.58	0.15–44.85
Subgaleal hematoma	0% (n=0)	0.4% (n=7)	3.96	0.22–71.11
Skull fracture with intracranial hemorrhage	0% (n=0)	0.3% (n=5)	5.4	0.29–100.26

## Discussion/comment

### Principal findings

In this retrospective cohort of 1606 forceps-assisted births, no statistically significant differences were observed in maternal or neonatal outcomes between rotational (Kielland’s) and non-rotational forceps deliveries. No cases of major neonatal cranial injury occurred in the rotational forceps group. Rates of postpartum hemorrhage were similar between groups, and although point estimates suggested lower rates of OASIS and major hemorrhage with rotational forceps, confidence intervals were wide and included the possibility of harm and benefit. Neonatal outcomes, including Apgar recovery, cord pH, and need for resuscitation, were comparable. These findings should be interpreted as descriptive, given limited statistical power and lack of adjustment for confounding. The absence of statistically significant differences in major maternal or neonatal outcomes should be interpreted cautiously, particularly given the relatively small number of rotational forceps deliveries and the retrospective nature of the study.

### Results in the context of what is known

These findings are consistent with contemporary literature suggesting that Kielland’s rotational forceps, when used by experienced operators, are not associated with increased maternal or neonatal morbidity as compared to nonrotational forceps deliveries and may help avoid second-stage cesarean delivery.^16^, ^17^, ^18^, ^19^, ^20^ Prior studies have demonstrated comparable or favorable outcomes for rotational forceps relative to alternative approaches, including reduced rates of OASIS and neonatal trauma in selected cases.^18^, ^19^, ^20^

The absence of major neonatal injury and similar short-term neonatal outcomes in this study align with reports demonstrating a reassuring neonatal safety profile for rotational operative vaginal delivery.^17^, ^18^, ^19^, ^20^, ^21^ However, as in previous studies, interpretation is limited by small sample sizes and potential selection bias, particularly as rotational forceps are often reserved for more complex cases involving fetal malposition.

The low utilization rate of rotational forceps (1.6%) reflects ongoing global declines in their use, attributed to medicolegal concerns, technical complexity, and reduced training opportunities.^17^, ^18^, ^19^ This trend has been well documented and contributes to variability in clinician experience and institutional practice patterns.

### Clinical implications

The decline in the use of forceps creates an important educational challenge: as the frequency of these procedures decreases, opportunities for trainees to acquire and maintain proficiency also decrease. The study findings suggest that Kielland’s rotational forceps may be a reasonable option for selected cases of second-stage labor arrest due to fetal malposition when performed by experienced clinicians. The comparable maternal and neonatal outcomes observed, despite use in more complex clinical scenarios, may reflect the effectiveness of appropriate patient selection and operator expertise.

A plausible explanation for the observed outcomes is that successful rotation to an occiput anterior position may reduce perineal trauma and facilitate vaginal birth, potentially avoiding the morbidity associated with second-stage cesarean delivery. However, given the observational design, small sample size, and likely confounding by indication, these results should not be interpreted as evidence of procedural equivalence or superiority.

From a clinical and training perspective, the findings highlight the importance of maintaining competency in rotational forceps techniques. Reduced procedural exposure and training opportunities have been associated with lower operator confidence and increased risk of adverse outcomes, including failed instrumental delivery and increased maternal morbidity.^22^^,^^23^^,^^24^ While simulation-based training may improve technical skills, it cannot fully replace clinical experience.^25^

This highlights the importance of maintaining competency in operative vaginal delivery and the potential role of structured simulation, supervised clinical training, and standardized educational approaches for rotational forceps techniques. Future research should examine how these skills can be effectively taught and maintained while ensuring appropriate patient selection and operator supervision.

Further evidence from adequately powered, prospective studies is required before these findings can be used to inform clinical guidelines or policy decisions.

### Research implications

Several important questions remain unanswered. Future research should consider operator experience and training background as important variables. Where feasible, prospective studies should document the operator’s level of experience with forceps, including experience specifically with rotational forceps, as this may be an important modifier of outcomes. Larger, multicenter prospective studies are needed to better evaluate the safety and effectiveness of rotational forceps, with adequate power to detect differences in uncommon but clinically significant maternal and neonatal outcomes. Future studies should incorporate adjustment for key confounders, including maternal characteristics, fetal position, indications for assisted vaginal birth (AVB), birthweight, and duration of the second stage of labor.

Inclusion of unsuccessful rotational forceps attempts, including those converted to cesarean delivery, is essential to provide a more complete assessment of patient safety and real-world outcomes. Subgroup analyses by fetal station and position may further clarify which patients are most likely to benefit.

Additional research is also needed to evaluate long-term maternal outcomes, including pelvic floor morbidity such as levator ani avulsion.^20^, ^21^, ^22^, ^26^ Studies assessing training strategies, including simulation and competency-based education, may help define optimal approaches to maintaining proficiency. Finally, qualitative research exploring clinician decision-making and institutional barriers may provide insight into the persistent underutilization of rotational forceps.

### Strengths and limitations

A key strength of this study is the inclusion of real-world clinical outcomes from a tertiary care setting over an extended study period, providing contemporary data on an under-studied obstetric procedure. The use of objective maternal and neonatal outcomes and a relatively large overall cohort enhances the descriptive value of the findings. Direct comparison with nonrotational forceps deliveries within the same institution provides important clinical context.

However, several limitations must be considered. Kielland’s forceps are likely to have been selected in circumstances in which the operator considered rotational operative vaginal delivery to be appropriate and feasible. Consequently, the observed outcomes cannot be interpreted independently of patient selection and operator judgment. The small number of rotational forceps cases (n=26) substantially limits statistical power and results in wide confidence intervals, precluding definitive conclusions. The absence of statistically significant differences should not be interpreted as equivalence.

The lack of adjustment for confounding due to incomplete data on key maternal and intrapartum variables introduces a high risk of confounding by indication, as instrument choice was nonrandom and likely influenced by clinical complexity. Additionally, exclusion of failed rotational forceps attempts converted to cesarean delivery may bias results toward more favorable outcomes and limits generalizability from a patient safety perspective.

The selective use of Kielland’s forceps may itself introduce selection bias. Operators may preferentially select patients in whom the procedure is considered technically favorable. Thus, the absence of increased adverse outcomes in our cohort should not be interpreted as evidence that Kielland’s forceps are appropriate for all patients requiring operative vaginal delivery.

Finally, differences in clinical context, including fetal position and station, limit direct comparability between groups. These findings are therefore best interpreted as hypothesis-generating and should be considered alongside existing literature.

## Conclusions

There has been declining familiarity with rotational forceps techniques among contemporary obstetric trainees and practicing obstetricians, particularly in settings where operative vaginal delivery has become less common. The findings of this study should not be extrapolated to inexperienced operators or interpreted as evidence that rotational forceps can be used safely without appropriate training, supervision, and patient selection. In this single-center retrospective cohort study, maternal and neonatal outcomes following rotational forceps delivery using Kielland’s forceps appeared similar to those observed following non-rotational forceps delivery. However, given the small sample size, lack of adjustment for confounding, and exclusion of unsuccessful operative vaginal birth attempts, these findings should be interpreted as descriptive and hypothesis-generating. Larger prospective studies are required before conclusions regarding safety, effectiveness, or training implications can be drawn.

## CRediT authorship contribution statement

**Paul Naseef:** Writing – review & editing, Writing – original draft, Methodology, Formal analysis, Conceptualization. **Caroline Leps:** Writing – review & editing, Software, Formal analysis. **Elham Almoi:** Software, Formal analysis. **Daniel Kane:** Writing – review & editing. **Nancy Kumar:** Writing – review & editing. **Melissa Walker:** Writing – review & editing. **John Kingdom:** Writing – review & editing. **Sebastian Hobson:** Writing – review & editing, Supervision, Conceptualization.
